# Attention and Default Mode Network Assessments of Meditation Experience during Active Cognition and Rest

**DOI:** 10.3390/brainsci11050566

**Published:** 2021-04-29

**Authors:** Kathryn J. Devaney, Emily J. Levin, Vaibhav Tripathi, James P. Higgins, Sara W. Lazar, David C. Somers

**Affiliations:** 1 Department of Neurology, Harvard Medical School, Boston, MA 02115, USA; 2 Department of Psychological & Brain Sciences, Boston University, Boston, MA 02115, USA; emily_levin@brown.edu (E.J.L.); vaibhavt@bu.edu (V.T.); hjames@bu.edu (J.P.H.); 3 Cognitive, Linguistic, and Psychological Sciences, Brown University, Providence, RI 02903, USA; 4 Department of Radiology, Northwestern University, Chicago, IL 60208, USA; 5 Department of Radiology, Harvard Medical School, Boston, MA 02115, USA; SLAZAR@mgh.harvard.edu

**Keywords:** meditation, attention, cognition, fMRI, dorsal attention network, ventral attention network, default mode network, resting-state functional connectivity, Vipassana

## Abstract

Meditation experience has previously been shown to improve performance on behavioral assessments of attention, but the neural bases of this improvement are unknown. Two prominent, strongly competing networks exist in the human cortex: a dorsal attention network, that is activated during focused attention, and a default mode network, that is suppressed during attentionally demanding tasks. Prior studies suggest that strong anti-correlations between these networks indicate good brain health. In addition, a third network, a ventral attention network, serves as a “circuit-breaker” that transiently disrupts and redirects focused attention to permit salient stimuli to capture attention. Here, we used functional magnetic resonance imaging to contrast cortical network activation between experienced focused attention Vipassana meditators and matched controls. Participants performed two attention tasks during scanning: a sustained attention task and an attention-capture task. Meditators demonstrated increased magnitude of differential activation in the dorsal attention vs. default mode network in a sustained attention task, relative to controls. In contrast, there were no evident attention network differences between meditators and controls in an attentional reorienting paradigm. A resting state functional connectivity analysis revealed a greater magnitude of anticorrelation between dorsal attention and default mode networks in the meditators as compared to both our local control group and a *n* = 168 Human Connectome Project dataset. These results demonstrate, with both task- and rest-based fMRI data, increased stability in sustained attention processes without an associated attentional capture cost in meditators. Task and resting-state results, which revealed stronger anticorrelations between dorsal attention and default mode networks in experienced mediators than in controls, are consistent with a brain health benefit of long-term meditation practice.

## 1. Introduction

Meditation—operationally defined as non-judgmental attention on the sensory experience of the present moment [1]—has previously been associated with improved performance on behavioral assessments of attention [2]. A separate line of investigation has shown increased activation, measured with blood oxygenation level dependent (BOLD) functional Magnetic Resonance Imaging (fMRI), in parietal regions during active meditation [3,4,5]. Since inefficient function of the attention system can have consequences ranging from the mundane (e.g., mind wandering) to the severe (e.g., traffic or surgical accidents), there is a high level of interest in methods to improve attentional efficacy. If meditation does produce generalized training of the attention system, we would expect experienced meditators to perform better than meditation-naïve participants in a variety of attention tasks. There is considerable literature demonstrating improvement on attention tasks in experienced meditators or in individuals who have received short-term meditation training [6,7,8,9,10], however, the putative neural correlates of these behavioral effects have not been demonstrated.

The human cortex comprises multiple interactive functional networks subserving distinct behaviors and states of consciousness. Two cortical networks serve attentional functions: (1) The Dorsal Attention Network (DAN), which includes the intraparietal sulcus (IPS), greater area MT (MT+), frontal eye fields (FEF) and the inferior branch of precentral sulcus (iPCS), supports sustained, focused attention [11,12,13,14,15,16]. (2) The Ventral Attention Network (VAN), which includes areas in the temporoparietal junction (TPJ) and inferior frontal gyrus (IFG), activates when a salient stimulus precipitates reorienting of attention (“Attentional Capture”) [11,17,18,19,20,21,22]. Additionally, another network, known as the Default Mode Network (DMN; including the posterior cingulate cortex, medial prefrontal cortex, anterior superior temporal sulcus, and angular gyrus) exhibits a reduction in activation during attentionally demanding tasks [23,24,25,26] and thus DMN deactivation is also a measure of attentional effects in the brain. Areas that co-activate during a particular task (i.e., the Dorsal Attention Network during a sustained attention task) are also co-active when the brain is at rest, making it possible to parcellate the brain into its component functional networks by correlating time courses of fMRI activation in resting state scans, where the participant is given no explicit task except to remain awake [27]. Employing this method, Yeo, Krienen et al., 2011 [28] suggest seven canonical networks that may be further parcellated into more precise and specialized functions [28,29,30]. 

The relationships between cortical networks vary over time, based on factors such as cognitive state [31] and age [32]. The magnitude of anticorrelation between DAN and DMN is a potential candidate marker for healthy aging and overall brain health [33]. For example, higher anticorrelation between default mode and dorsal attention network predicts greater success on cognitive assessments of attention [34] and decreased gait variability and thus risk of falls in elderly adults [35]. Greater correlation (i.e., weaker anticorrelation) between these two networks, when controlling for the effects of age, predicts cognitive impairment [36] and self-reported attitudes of anger and aggression [37]. Meditation experience has previously been shown to suppress default mode network structures at rest [38] and mitigate age-related cortical thinning [39], and functional connectivity between DMN and frontal cortical areas has been shown to be weaker at rest in experienced, relative to novice, mindfulness meditators [40]. These findings taken together suggest that a relative down-regulation of the DMN and up-regulation of other networks is an effect of meditation training that could lead to improved brain health, increased happiness [41], and positive aging outcomes. 

Three broad categories of meditative practice have been proposed [42,43]: focused attention methods (such as Vipassana and Shamatha, which encourage vigilant focus on the object of meditation, which is often the breath), loving-kindness methods (such as metta, in which happiness is wished upon all beings), and receptive, open-monitoring methods (in which thoughts and sensations are observed without judgment). Burmese Vipassana meditation is a commonly practiced focused attention (FA) meditation technique, and involves sustaining attention for an hour or more on subtle physical sensations while filtering out any endogenous and exogenous distractions [44]. A typical Vipassana training retreat is 10 days long, during which time students meditate for 10–12 h per day in 45-min to two-hour increments. When attention drifts or is captured by some feature of the environment, students are encouraged not to dwell on the distraction or on the lapse of attention, but rather to calmly bring focus back to the breath and continue to hold attention there. Over time, this becomes progressively easier. As the task demands of focused attention meditation require sustaining attention and repeatedly refusing to reinforce reorienting signals generated in response to distractors, focused attention meditation methods can be viewed as a systematic effort to improve sustained attention.

The work reviewed above indicates that meditation experience correlates with improved outcomes on behavioral assessments of attention, and that the meditative state correlates with activation in cortical attention networks. Thus, two converging lines of evidence demonstrate that meditation experience is associated with improved performance on sustained attention tasks behaviorally, and with altered activation in cortical attention networks. A logical next step is to bridge these two lines of evidence by quantifying activation in cortical attention networks while meditation practitioners perform sustained attention tasks. 

Here, we sought to examine attentional function in highly experienced focused attention meditators while they were performing highly demanding yet non-meditative attention tasks. Rather than describe spatiotemporal patterns of cortical activity related to a mediative state, we treat focused attention meditation as a trait-level training task for attention itself, and measure outcomes of engaged attention in attentional and default mode networks. Since training effects should be most evident in those individuals who have had a great deal of training, we recruited meditators with thousands of hours of meditation experience across many years. We contrasted the meditators with a group of control subjects, matching for age and education level as well as for demand characteristics of the experiment (see Methods). We examined behavioral performance and fMRI activation across groups in two visual attentional tasks. The Multiple Object Tracking (MOT) task [45,46] was employed as the sustained visual attention task and was intended to examine the Dorsal Attention Network (DAN), which is associated with self-directed attention, as well as the Default Mode Network (DMN), which is suppressed during performance of attentionally demanding tasks. In a second task (ViNO), vivid, novel oddball distractor stimuli were presented during the performance of the attentionally demanding task [47] in order to examine the Ventral Attention Network (VAN), which is associated with stimulus-driven attention or attentional capture effects. We hypothesized that (1) experienced meditators would exhibit an attentional benefit in performance of a sustained attention task; (2) experienced meditators would more effectively filter out task-irrelevant stimuli in an attentional capture paradigm; (3) experienced meditators would exhibit greater opponency between activation of the Dorsal Attention Network and deactivation of the Default Mode Network during performance of a sustained attention tasks; and (4) experienced meditators would exhibit less activation of the Ventral Attention Network during an attention capture paradigm. In order to further investigate group-level differences in the opponency of DAN and DMN, we examined resting-state functional connectivity in experienced mediators, our in-lab control subjects, and a large (*n* = 168) cohort of subjects from the Human Connectome Project dataset.

## 2. Materials and Methods

### 2.1. Participant Recruitment

Sixteen experienced meditators (11 male, mean age = 34.33 years, mean education = 15.2 years) were snowball sampled (i.e., we allowed participants to recruit other participants via word of mouth, see [48,49]) from a Vipassana meditation center in Massachusetts, starting from a recruitment letter that we emailed and hand-delivered to key center personnel. Each meditator completed a set of “Meditation Demographic Forms” detailing their lifetime experience with meditation as well as their age, gender, education level, handedness, and number of languages natively spoken. These demographic criteria were used to recruit one unique control subject for each individual meditator. Control subjects were matched on five demographic criteria (age +/− 1 year, gender, education level +/− 2 years, handedness, and number of languages natively spoken). Ultimately, 30 participants, 16 meditators and 14 matched control subjects, were recruited. At a group level, the controls closely matched the meditators (control group mean age = 34.33 years, mean education = 14.9 years). All subjects were compensated and gave written informed consent to participate in the study, which was approved by the Institutional Review Board of Boston University (1040E, 2734E).

Because the meditators were aware that they were being recruited specifically due to their meditation experience, which has the potential to alter their performance on the task, control subjects were recruited on the basis of “sham expertise” in order to create an equal expectation of high performance in the controls [50]. Each potential control subject determined to be a match on the five demographic criteria of interest completed a survey asking for their expertise level on a five-point Likert scale (far below average, below average, average, above average, far above average) in a number of areas (e.g., driving, recognizing faces, athletic ability). In the event that recruits rated themselves at or below average on all areas, there was also an open-ended section of the form where they were required to fill in one talent or ability at each of the five levels of performance. An item on which the potential control rated themselves as “far above average” was substituted for “meditation” in the Meditation Demographic Form and subsequently filled out by the control subject (e.g., a control participant who rated themselves “far above average” at swimming would be given a “Swimming Demographic Form” containing all the same questions as the Mediation Demographic Form). We refer to these subjects as “sham-expertise controls”.

Participants recruited as meditators (*n* = 16) had an average of seven years of meditation experience (standard deviation ±6 years, range = 1–20). The average reported meditation sessions per week was 14 (±4; range = 9–26) with 60 min (±2 min; range = 55–65) reported per session, for an average of 843 min. (±230; range = 540–1560) per week of meditation. The average number of estimated lifetime hours of meditation experience was 8311 (±11,682; range = 1300–50,000). The average number of days spent on silent meditation retreats was 291 (±86; range = 25–319). 

For the resting-state fMRI analysis, in order to overcome subject attrition in the sham-expertise control group (*n* = 8), we included 13 additional control subjects (eight female, age range 24–38, mean education > 16 years) from the Boston University community to form a group of 21 “in-lab” control participants. All participants were financially compensated ($75 per hour) and gave written informed consent to participate in the study, which was approved by the Institutional Review Board of Boston University. A second control group was drawn from the publicly available Human Connectome Project (HCP) dataset [51]. The HCP control group (*n* = 168) consisted of the anonymized cohort of healthy subjects (64 male, 104 female; age ranges: 22–35 *n* = 18, 26–30 *n* = 82, 31–35 *n* = 65, 36+ *n* = 2) that had participated in both 3T and 7T studies (db.humanconnectome.org; accessed on August 26, 2019). See Appendix A for HCP subject codes.

### 2.2. Attention Tasks Performed during fMRI Scanning

Participants were presented with two demanding visual attention tasks inside of the fMRI scanner: the multiple object tracking (MOT) task [45,46] and a vivid, novel oddball (ViNO) paradigm previously developed as a Ventral Attention Network localizer [47]. The order of tasks (MOT, ViNO) was counterbalanced across subjects. In addition, subjects were scanned in the resting state, with no explicit task. Subjects with meditation expertise were explicitly instructed not to meditate during the resting state scans. We did not formally interview the meditators post-scan about their ability to maintain a non-meditative state during the resting state scans; however, in the pre-scan instructions, they were specifically instructed not to meditate and not to “exert any conscious control over [their] mental state” with time after that instruction for questions and clarification.

**Figure 1:**
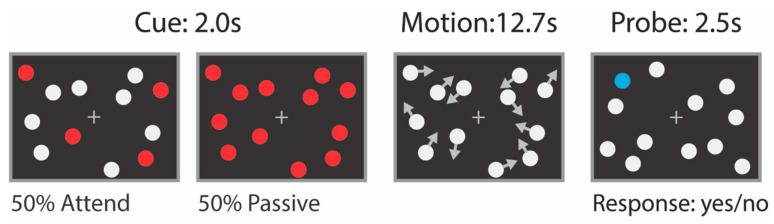
The Multiple Object Tracking (MOT) Task. The MOT task is designed to localize areas of the cortex responsible for sustained, focused attention (i.e., the dorsal attention network). Participants fixate a central cross for the duration of the run. In “attend” trials, subjects are asked to track the location of four discs that are marked red during the cueing period. All discs then turn white and begin to move smoothly on the screen. During “attend” trials, participants must keep track of the four initial red (now white) discs as they move. Following 12.7 s of motion, discs are once again stationary, and one “probe disc” turns blue. During “attend” trials, participants indicate (yes/no) whether the probe corresponds to one of the four discs which they were tracking. During “passive” trials, all 12 discs are red during the cueing period, and there is no attentional demand during the motion period aside from maintaining central fixation. During “passive” trials, participants make a random button press at the appearance of the probe. Thus, all bottom up visual and top-down motor processes are controlled across trials, while only attention is manipulated.

Multiple Object Tracking (MOT) Task: Participants were required to sustain attention to moving objects (Figure 1). MOT trial duration was 18.2 s (seven 2.6 s TRs). Each trial in the MOT task consisted of three phases: cue (3 s), motion (12.7 s), and probe (2.5 s). During the cue phase, participants viewed 12 stationary white discs (restricted to left and right portions of the screen by two invisible rectangular barriers with six discs per visual hemifield), and four of the discs (two per hemifield) briefly flashed red before returning to white. The participants’ task was to hold central fixation while tracking the location of these four flashed discs. After the red discs returned to being colored white, all 12 discs began to smoothly drift around on the screen. The cue period timing was as follows: a pre-cue period (12 stationary white discs on the screen) of 0.5 s, a cue period (discs are colored red) of 2.0 s, and a pretrial period (again 12 stationary white discs on the screen) of 0.5 s. The discs moved on independent trajectories and were programmed with a repulsion algorithm [52], which operates between each and every disc plus the rectangular “walls” constraining each disc to its hemifield, so that discs did not overlap or leave their assigned hemifield. After 12.7 s, the discs ceased moving, and one single probe disc turned blue. The participant then indicated whether the probe disc was included in the set of four discs that they were tracking (2AFC yes/no). Each of these “attend” trials was interleaved with “passive” trials in which all 12 discs initially flashed red, and participants were required only to maintain central fixation and make a button press of their choosing at the end of the trial. 

Since the visual stimulation was equated across the “active” and “passive” conditions, the contrast of “attend” versus “passive” allows us to distinguish DAN structures independent from cortical areas primarily concerned with bottom-up stimulus processing. “Attend” trials are expected to yield stronger activation than “passive” trials within areas in the dorsal attention network [15,53]. Each run was 16 trials (7TRs per trial, 2.6 s TR) plus 2TRs of fixation at the beginning and end, for a total of 116 timepoints per run. Each participant performed one practice run outside of the scanner and four runs (464 total timepoints) inside the scanner.

**Figure 2:**
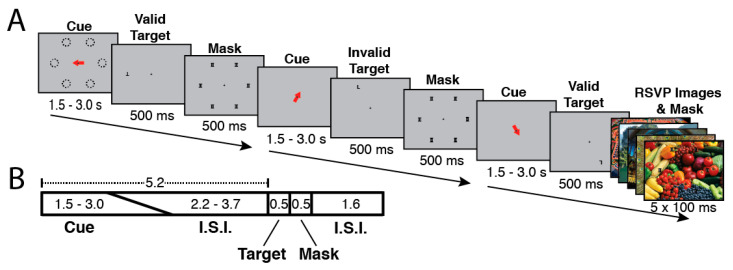
ViNO Task Trial Structure and Timing. The Vivid Novel Oddball (ViNO) task is designed to examine Ventral Attention Network structures by combining both spatial attentional reorienting and stimulus-driven attentional capture. (**A**) Examples of each of three trial types (Valid, Invalid, Oddball). Each trial began with a spatial cue indicating one of six possible locations (dashed lines in (**B**), dashed lines were not visible during the experiment). Following an inter-stimulus interval (I.S.I), the target appeared. The target was the letter “T” or “L” and could appear upright or inverted. The cue could be valid (target appears at the cued location) or invalid (appears in an uncued location). Following the target, a mask of superimposed “T’s and “L”s was presented at all possible target locations. On a randomly selected 1/6 of trials, a task-irrelevant RSVP stream of five vivid, full-screen, session-unique “oddball” distractor images appeared behind the letter mask, each with 100 ms duration. (**B**) Trial Timing: The orienting phase was 5.2 s in total, with cue duration randomly jittered from 1.5–3.0 s and the fixation cross ISI accounting for the remaining 5.2 s. Following the orienting phase, the target appeared for 500 ms, followed by the mask or oddball mask for 500 ms. The mask/oddball distractor phase was followed by an inter-trial interval of 1.6 s. Participants responded to indicate the identity of the target letter (T or L).

Vivid, Novel Oddball (ViNO) Paradigm: Participants performed a sustained spatial attention task that was interrupted by infrequent oddball distractor events in the form of colorful, novel, full-screen images (Figure 2). The goal of this task was to engage subjects in a focused attention task and then disrupt and reorient attention to task-irrelevant stimuli. Vivid novel oddball stimuli were used to create compelling stimulus-driven attentional capture events that would trigger activation of the ventral attention network. 

Each trial lasted 7.8 s and consisted of the following phases: orienting phase (cue and ISI), target phase (letter on screen), mask phase (mask on screen), and ITI (fixation cross on screen). During the orienting phase, a red arrow cue appeared and indicated one of six possible target locations on the screen. To encourage attention to only the cued location, participants were instructed in advance that the letter target would appear at the spatially cued location “more often than not”, and that the letter target would never appear at a location other than the six possible cued locations. The timing of the orienting phase was jittered such that the duration of the cue and ISI together always summed to 5.2 s, with the cue taking a random value between 1.5–3.0 s and the ISI filling the rest of the 5.2 s; the unpredictable interval from cue offset to target onset encouraged the deployment of sustained attention over the full ISI. Following the orienting phase, the target (upright or inverted T or L) appeared for 500 ms either at the cued (valid) or an uncued (invalid) location. Invalid targets always appeared in a random location (one of three) in the uncued hemifield. Spatial cues were valid on 68.75% of trials (33 out of 48 trials per run). Participants responded with one of two keys on a button box to indicate the identity of the letter target regardless of its orientation or location. The target was followed by a 500 ms mask of superimposed T’s and L’s at all of the six possible target locations (Figure 2). Following the mask, participants were shown a fixation cross for the ITI of 1.6 s. Responses were recorded from target onset to the start of the following trial (a 2.6 s total possible response period). Each run consisted of six blocks of eight trials each (48 trials/run), and participants completed four runs per scan session.

In each run, on 1/6th of total trials (randomly selected), the post-target mask was displayed with a background of five full-screen (approximately 22 × 16.5 degrees), full-color, unique “oddball” distractor images each presented for 100 ms (Figure 2). Oddball distractors were presented in a “rapid-fire” or rapid serial visual presentation (RSVP) burst (five images, 100 ms per image). The intention of the “rapid-fire” oddball sequence was to produce a strong, unexpected attentional capture effect. During four fMRI scan runs, participants saw 192 total trials including 32 oddball trials (160 unique oddball images). Because prior work [54,55] indicates the presence of a visually responsive face-processing region in posterior superior temporal sulcus, images with prominent human faces were excluded from the oddball distractor image set. Each run also included 7.8 s of fixation (no stimuli other than a fixation cross) at both the start and the end of the run. Each participant was scanned on 192 total trials (including 32 total oddball trials), plus one practice run with trial-wise performance feedback outside of the scanner for training. All participants received instructions to respond as quickly as possible without sacrificing accuracy, and were given an opportunity to ask questions both before and after completing the practice run. The practice runs did not contain any oddball images and participants were not informed that oddball images would occur, only that they should indicate the identity of the target letter despite variations in its location or orientation, or any other stimuli that might occur.

### 2.3. MRI Scanning Parameters

All task MRI data and all in-lab resting-state MRI data were acquired using a 32-channel Siemens head coil in a horizontal bore 3 Tesla Siemens Tim Trio located at the Harvard University Center for Brain Science in Cambridge, MA. Gradient echo EPI sequences were used for all tasks (TR = 2600 ms, TE = 30 ms, Flip angle = 90°, voxel size = 3.0 × 3.0 × 3.1 mm, 42 slices, whole brain coverage). Magnetization Prepared Rapid Gradient Echo (MP-RaGE) T1-weighted high-resolution data (TR = 6.6 ms, TE = 2.9 ms, Flip angle = 8°, voxel size = 1.0 × 1.0 × 1.3 mm) were acquired for each participant on the same scanner as the task data. In-lab resting-state data consisted of two runs of 6 min each (278 timepoints). The Human Connectome Project (HCP) resting-state data consisted of four runs of fifteen minutes of resting state data per subject. The runs were normalized across time and concatenated to give us sixty minutes of resting state data per subject.

### 2.4. Data Analysis

Cortical reconstruction and volumetric segmentation of the T1 data was performed with the Freesurfer image analysis suite, which is documented and freely available for download at http://surfer.nmr.mgh.harvard.edu/. The technical details of these procedures are described in prior publications [56,57,58,59,60,61,62,63,64,65]. All task data were analyzed with fs-fast version 5.1.0 and custom Matlab scripts. Data were first slice-time corrected and motion corrected. For the task data, motion correction, volumetric spatial smoothing (hwhm = 1.5 mm), intensity normalization, and boundary-based registration [66] to the participant’s own anatomical data were performed on a per-run basis. Singular value decomposition reduced the six motion correction vectors to three eigenvectors, which were included as nuisance regressors in the model. 

Analyses were performed separately in each hemisphere on each subject’s own cortical surface, and data were analyzed for each vertex using a GLM with each condition as a predictor (i.e., in ViNO, one each for Valid Target, Invalid Target, Oddball, Non-Oddball, and Fixation and in MOT, one for Attend, Passive, and Fixation). The BOLD signal was modeled with a γ response function assumed for each condition with a delay δ = 2.25 and a delay time constant τ = 1.25. An estimated response was generated by convolving the response function with the event length (i.e., the time in each condition) and minimizing the residual error (FS-FAST). A *t*-test was performed for each vertex to compare differences in activation between conditions. The significance of these activation differences was visualized on the surface of each subject’s own hemisphere. The spatial cueing analysis was conducted with the contrast of Invalid vs. Valid Targets, and compared with regions activated for oddball stimuli with the Oddball vs. Non-Oddball contrast. The MOT analysis was conducted with the contrast of Attend vs. Passive.

For the group average analysis, each participant’s fMRI data were registered to an average cortical surface space (Freesurfer 5.1.0, “fsaverage” brain) using the boundary of the gray matter and white matter [66]. The GLM analysis methods were the same as for individual subject data; however the significance of these activation differences was computed on vertices of the fsaverage brain, and visualized on that surface. We then employed a random-effects model to compute the group-averaged value for each condition at each vertex before running *t*-tests at each vertex to compare group-level activation differences per condition. Significant group-level task activation was corrected for multiple comparisons using cluster-based correction [67,68], permuting the sign on the design matrix (using the FS-FAST tool mri_glmfit-sim) with cluster thresholding at a corrected alpha of *p* < 0.01.

Behavioral responses and spatio-temporal patterns of neural activation were directly compared with meditation-naïve humans matched for age, gender, education level, handedness, and number of languages natively spoken. In addition to constructing within-group averages for the meditation group and the control group separately, a between-group whole brain independent samples t-test was conducted at each vertex to directly compare active regions between the groups for the oddball > non-oddball and invalid > valid contrasts of the VAN localizer, and the attend > passive contrast of MOT. Regions of Interest (ROIs) were defined using the Yeo and Krienen [28] functional atlas projected onto each individual hemisphere. Percent signal change for each ROI for each contrast was obtained with the fs-fast funcroi-table tool, which computes the mean percent signal change over all vertices within a region of interest for a specified contrast. All ROI analyses were conducted at the individual subject level within fs-fast and then compared between groups. 

ANOVAs were conducted using a linear mixed effects model, which nested the random effects of individual subjects to examine the effect of group (meditator, non-meditator) and hemisphere (rh, lh) on the percent signal change within each ROI (including interaction terms) implemented with the lmer function in R. Effect sizes were calculated using Cohen’s d with pooled standard deviations. Pearson correlations between ROI data and behavioral and demographic data were computed using the “corr” tool in MATLAB [69].

Resting-state data were preprocessed in MATLAB. Head-motion regression (six motion parameters and their six temporal derivatives), whole-brain signal regression, and ventricular and white matter signal regression were performed [70]. We then calculated framewise displacement by taking the sum of the absolute derivatives of the six motion parameters for each time point [71]. A threshold of 0.5 mm was set to identify time points with excessive motion. To avoid artifact spread during bandpass filtering, high motion time points were replaced using linear interpolation [72]. Band-pass filtering was then performed to extract frequencies between 0.01 and 0.08 Hz. After filtering, high-motion time points were removed. The resting state runs were normalized across time and concatenated. We then regressed out the mean signal and used it in the connectivity analysis. Resting-state functional connectivity analysis was performed using the Schaefer 100 parcel cortical atlas [73], which was arranged into seven Yeo Networks [28]. Default Mode Network and Dorsal Attention Network resting state data were averaged across time and then correlated using Pearson correlation separately for the right and left hemispheres. The matched control dataset was combined with the in-lab control dataset on thirteen subjects acquired on the same scanner, which resulted in three datasets: meditator dataset, in-lab control dataset, and HCP dataset. In order to match the number of time points in the resting state data, across datasets, which were used to compute connectivity, we selected the first 250 TRs across the three datasets, and a single value per hemisphere per subject was computed. The connectivity was then compared across the three datasets using independent samples t-test as implemented in the SciPy Python toolbox [74] and two way ANOVA using the statsmodels toolbox [75].

## 3. Results

### 3.1. Multiple Object Tracking: Behavior

Behavioral performance on attend trials (Table 1) measured by Cowan’s *k* score [76] (*k* = (Hit Rate–False Alarm Rate) × 4.0) was not significantly different between the experienced meditator group (mean *k* = 1.25) and the “sham expertise” control group (mean *k* = 0.82, *t*_(26)_ = 1.10, *p* = 0.28). There were no group differences in hit rate (meditator mean = 0.64, control mean = 0.57; *t*_(26)_ = 0.99, *p* = 0.33) or false alarm rate (meditator mean = 0.33, sham-expertise control mean = 0.39, *t*_(26)_ = −0.75, *p* = 0.46). 

### 3.2. Multiple Object Tracking: fMRI Activation

**Figure 3:**
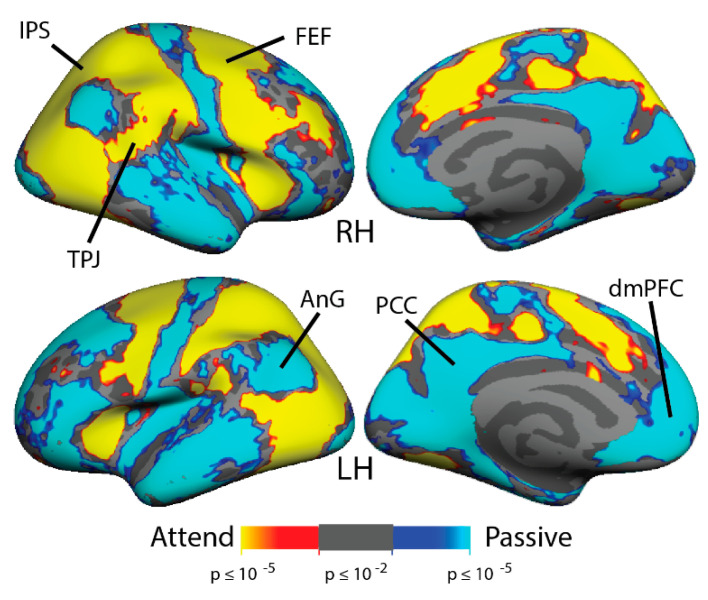
Multiple Object Tracking Localizes the Dorsal Attention Network in a Cross-Group Average (all subjects). The MOT group average, containing 16 meditators and 12 non-meditators for the “attend vs. passive” trials, localizes Dorsal Attention Network areas (including Intraparietal Sulcus [IPS] and Frontal Eye Fields [FEF]) and deactivates the Default Mode Network (including the Posterior Cingular Cortex [PCC], dorsomedial Prefrontal Cortex [dmPFC], and Angular Gyrus [AnG]). RH = Right Hemisphere, LH = Left Hemisphere, TPJ = Temporoparietal Junction.

**Figure 4:**
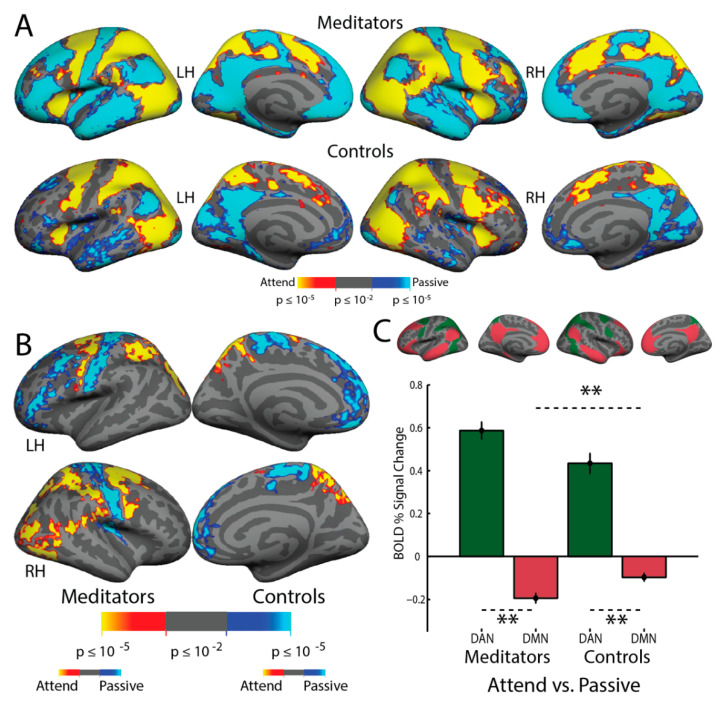
Meditators and non-meditators demonstrate different levels of activation in the Dorsal Attention Network and deactivation in the Default Mode Network while attending. (**A**) Within-group averages for the meditators (*n* = 16) and non-meditators (*n* = 12) for the “attend vs. passive” contrast of the Multiple Object Tracking task, shown at the same threshold (min = *p* ≤ 0.01, max = *p* ≥ 0.00001). Meditators display greater spatial extent of activation in the Dorsal Attention Network and more apparent deactivation in the Default Mode Network when paying attention. (**B**) Between-group cluster-corrected fixed effects average of the meditators vs. the non-meditators for the “attend vs. passive” contrast. Meditators demonstrate significantly more activation in all nodes of the Dorsal Attention Network (MT+, IPS, FEF, iPCS) and more deactivation in the Default Mode Network (inferior temporal cortex, angular gyrus). (**C**) Top: the Dorsal Attention Network and Default Mode network ROIs used in the analysis-each set of network ROIs were projected onto each individual hemisphere. Bottom: Both groups demonstrate significantly higher levels of activation in the Dorsal Attention Network and deactivation in Default Mode Network while attending, with the meditators (left) showing greater suppression in DMN than the non-meditators (right). Error bars depict standard error of the mean; ** = *p* < 0.05 and effect size > 0.8 (see Results for details).

In order to observe the overall pattern of brain activity in the Multiple Object Tracking task, the attention and passive stimulation conditions were contrasted in each subject and were averaged across all subjects (*n* = 28). Figure 3 demonstrates the expected activation of the Dorsal Attention Network (including cortical areas MT+, IPS, FEF, and iPCS) and deactivation of the Default Mode Network (including posterior cingulate cortex, medial prefrontal cortex, anterior superior temporal sulcus, and angular gyrus). In a within-groups analysis conducted for the attend vs. passive contrast separately in the meditators and non-meditators (Figure 4A), meditators displayed more evident activation in dorsal attention network areas and more evident default mode network deactivation than non-meditators. In the group average, meditators also displayed a patch of activation in the fundus of the superior temporal sulcus in the temporoparietal junction while attending that was not evident in non-meditators. In a between-group full-brain GLM comparison (Figure 4B), the meditators demonstrate significantly greater activation in MT+, the IPS, the FEF, and the anterior insula (AI). They also demonstrate greater suppression while attending in the angular gyrus, motor cortex, and left lateralized inferior frontal sulcus. Moreover, the meditators display more activation while attending in the RH TPJ and the LH Supramarginal Gyrus (SMG), which are both nodes of the Ventral Attention Network. 

### 3.3. Multiple Object Tracking: Region of Interest Analyses

We conducted ROI analysis using the Yeo–Buckner Atlas definitions [28] of the Dorsal Attention Network (DAN) and the Default Mode Network (DMN). The difference in DAN vs. DMN activation while attending was significantly larger in the meditators than in sham-expertise controls (*F*_(1, 26)_ = 6.48, *p* < 0.05, mean meditator difference = 0.7808, mean control difference = 0.5307, effect size = 0.88). There were no effects of hemisphere (*F*_(1, 26)_ = 2.29, *p* = 0.14) or interaction effects (*F*_(1, 26)_ = 0.01, *p* = 0.93). These results quantify the greater activation in DAN and suppression in DMN evident in the full-brain between-group average results (Figure 4). Examination of only the DAN revealed only a trend-level group effect, despite a medium effect size (*F*_(1, 26)_ = 3.4, *p* = 0.08, mean DAN meditators = 0.5870, mean DAN controls = 0.4342, effect size = 0.65). Across all subjects, a significant laterality effect was observed, with greater BOLD signal change in the right hemisphere of the DAN (*F*_(1, 26_) = 5.04, *p* < 0.05, mean RH = 0.5750, mean LH = 0.4680, effect size = 0.45). There was no group x hemisphere interaction (*F*_(1, 26)_ = 0.36, *p* = 0.56). A significant effect of group was observed in the DMN, with the meditators suppressing DMN significantly more than controls while attending (*F*_(1, 26)_ = 6.24, *p* < 0.05, mean meditators = −0.1938, mean non-meditators = −0.0965, effect size = 0.82). There were no effects of hemisphere in the DMN (*F*_(1,26)_ = 2.16, *p* = 0.16) and no interaction (*F*_(1, 26)_ = 0.52, *p* = 0.48).

### 3.4. Relationship between Behavior, Meditation Experience, and BOLD Activation

Within subjects, the magnitude of the DAN–DMN difference was significantly correlated with *k* score overall (RH *r* = 0.53, *p* < 0.01; LH *r* = 0.47, *p* < 0.05), and this relationship was driven by the control subjects (control RH *r* = 0.72, *p* < 0.01; LH *r* = 0.74, *p* < 0.01; meditator RH *r* = 0.41, *p* = 0.11; LH *r* = 0.30, *p* = 0.26). In the examination of each group, this correlation was significant in the right hemisphere only (meditators rh *r* = 0.54, *p* < 0.05; controls rh *r* = 0.61 *p* < 0.05; meditators lh *r* = 0.36, *p* = 0.17; controls lh *r* = 0.53, *p* = 0.07). DMN suppression was not correlated with *k* score overall (rh *r* = −0.08, *p* = 0.68; lh *r* = −0.32, *p* = 0.10). DMN suppression and *k* score were significantly related in the controls (control rh *r* = −0.73, *p* < 0.05; lh *r* = −0.84, *p* < 0.001), but not in the meditators (rh *r* = 0.27, *p* = 0.32; lh *r* = −0.38, *p* = 0.89). DAN activation and *k* score were also significantly correlated (*r* = 0.52, *p* = 0.0045), and this relationship was stronger in the control group (meditator *r* = 0.48 *p* = 0.062; control *r* = 0.58 *p* = 0.047).

Since prior reports have indicated relationships between meditation experience and activation in attention networks while in a meditative state (e.g., [5]), here we examined whether prior experience with meditation demonstrated any relationship with the magnitude of activation in the DAN while attending, or magnitude of the DAN–DMN difference. We used years of meditation practice, days spent on meditation retreats, and minutes per week of meditation as our metrics of experience. Amongst the meditators, the magnitude of activation in the DAN while attending was not significantly correlated with years of meditation practice (mean years = 7.01; *r* = −0.04, *p* = 0.8) or with days spent on meditation retreats (mean retreat days = 291, *r* = −0.20, *p* = 0.45). A trend correlation was observed between DAN activation and minutes per week of meditation (mean = 843; *r* = 0.36, *p* = 0.06), suggesting that recent mediation experience may have a larger influence on DAN activation while attending than lifetime meditation experience does.

### 3.5. ViNO: Behavior

The ViNO task (Figure 2) combines a spatial cueing task that contains occasional invalid cues with the unpredictable presentation of novel oddball distractors. In prior work, we have demonstrated that this task effectively drives regions of the Ventral Attention Network [47]. Reaction time and accuracy were measured for the identification of target letters (T or L) in valid and invalid cueing conditions. Additionally, the influence of oddball distractors was examined. One meditation participant exited the scanner due to discomfort prior to commencing the ViNO task. For the remaining 15 meditation and 12 control subjects, there were no differences in accuracy between groups (meditation mean = 87.30%, control mean = 87.93%, *t*_(25)_ = −0.2581, *p* = 0.80). 

As expected, overall reaction time for invalid targets was significantly longer than reaction time for valid targets (mean valid = 801 ms, mean invalid = 851 ms, *t*_(__26)_ = 5.4, *p* < 0.01). The magnitude of the difference between invalid and valid targets was not different between the groups (invalid vs. valid mean difference in meditators = 42 ms, in controls = 57 ms, *t*_(25)_ = −0.82, *p* = 0.42). Meditators displayed significantly longer reaction times than controls on valid trials (125 ms longer, *t*_(25)_ = 2.27, *p* < 0.05) and a trend towards longer reaction times on invalid trials (110 ms higher, *t*_(25)_ = 1.76, *p* = 0.09). The slower overall responses of meditators is not easily explained; however, it is consistent across trial types. There was no speed-accuracy tradeoff in this dataset (speed-accuracy correlation *r* = −0.0798, *p* = 0.7), and there was no relationship between age and RT (*r* = 0.27, *p* = 0.17).

There was a positive relationship between years of meditation experience and reaction time, with more years of meditation experience predicting longer reaction times (*r* = 0.58, *p* < 0.05), even though there was no relationship between age and reaction time. No relationship between average minutes per week of meditation and reaction time (*r* = −0.14, *p* = 0.61) or retreat days and reaction time (*r* = 0.31, *p* = 0.12) was observed. There was no relationship between meditation experience and accuracy (years *r* = 0.32, *p* = 0.24; minutes per week *r* = 0.31, *p* = 0.27; retreat days *r* = 0.25, *p* = 0.22). 

### 3.6. ViNO: fMRI Activation

**Figure 5:**
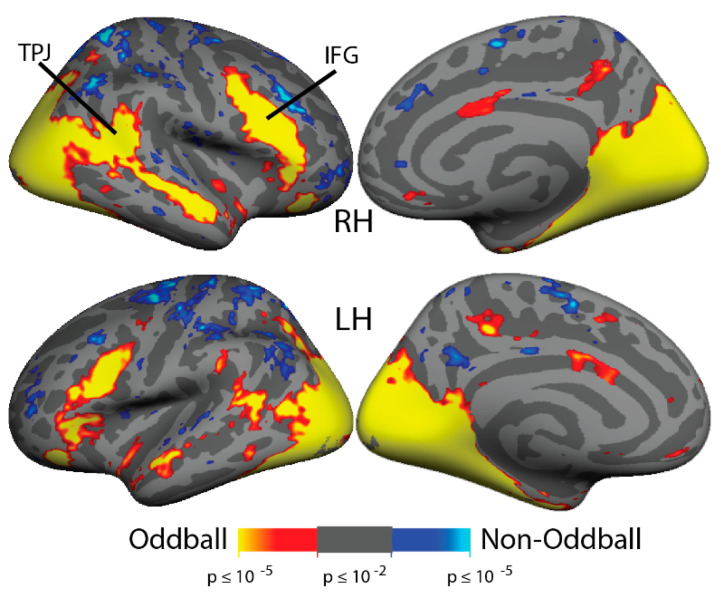
ViNO Task localizes Ventral Attention Network in a group average. The ViNO cross-group average, containing 15 meditators and 12 non-meditators for the “oddball vs. non-oddball” contrast, localizes the temporoparietal junction (TPJ) and the inferior frontal gyrus (IFG) of the Ventral Attention Network.

**Figure 6:**
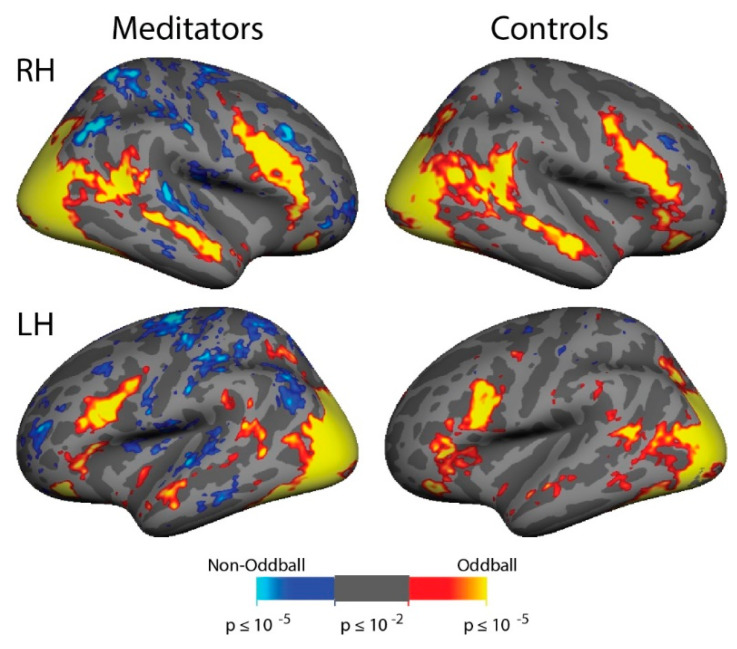
Meditators and non-meditators display similar cortical patterns of activation during bottom-up attentional capture. Within-group averages for the meditators (*n* = 15) and the non-meditators (*n* = 12) for the “oddball vs. non-oddball” contrast of the ViNO task, shown at the same threshold (min = *p* ≤ 0.01, max = *p* ≥ 0.00001).

**Figure 7:**
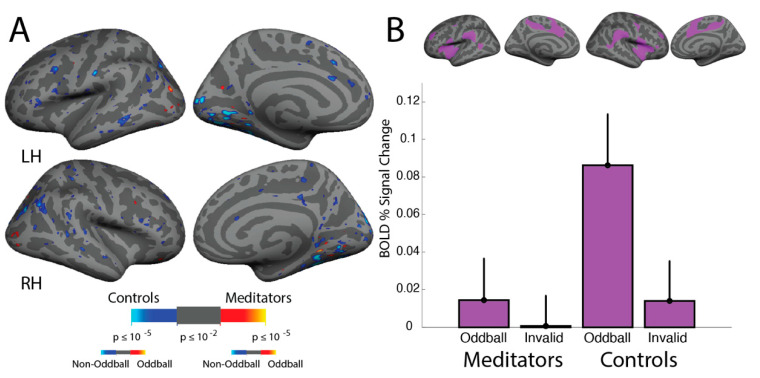
Oddball Effects in VAN in meditators and non-meditators. (**A**) In a between-group fixed effects average of the meditators vs. the non-meditators during attentional capture, as reflected in the “oddball vs. non-oddball” contrast, no group differences are evident in the full brain between group average. (**B**) Top: The ventral attention network of the Yeo, Krienen et al. parcellation used to compute BOLD percent signal changes in each individual subject. Bottom: In an ROI analysis including only vertices in the Yeo, Krienen et al. [28] parcellation of the Ventral Attention Network, the meditators and controls demonstrate no difference in the Ventral Attention Network for spatial reorienting or oddball appearance (error bars depict standard error of the mean).

In a group average of all participants pooled together (*n* = 27), the “oddball vs. non-oddball” contrast of the ViNO task activates Ventral Attention Network structures (Temporoparietal junction and inferior frontal gyrus) (Figure 5). Both the meditators and matched controls demonstrated a similar pattern of cortical activation for oddball trials relative to non-oddball trials (Figure 6); each group displayed activation in the temporoparietal junction, inferior frontal gyrus, and anterior insula, as was expected for this task. A full-brain between-group average revealed no differences between the groups (Figure 7A).

### 3.7. ViNO: ROI Analyses 

An ROI analysis was conducted in the Yeo, Krienen et al. (2011) [28] Ventral Attention Network (VAN) in the meditators and non-meditators, for both spatial reorienting (invalid vs. valid cue) and for oddball (vs. non-oddball) trials separately. There were no spatial reorienting effects (Figure 7B) by group (*F*_(1, 25)_ = 0.16, *p* = 0.69, meditator mean = 0.000, non-meditator mean = 0.0140, effect size = 0.13) or by hemisphere (*F*_(1,25)_ = 2.16, *p* = 0.15) and no interaction (*F*_(1, 25)_ = 1.77, *p* = 0.20). There were also no oddball effects by group (*F*_(1, 25)_ = 2.05, *p* = 0.16, meditator mean = 0.0144, control mean = 0.0862, effect size = 0.13; Figure 7B) or hemisphere (*F*_(1, 25)_ = 0.22, *p* = 0.64) and no interaction (*F*_(1, 25)_ = 0.003, *p* = 0.95).

Isolating the TPJ ROI, there were no effects of cue validity between groups (*F*(1, 25) = 0.002, *p* = 0.96, meditator mean = 0.0044, control mean = 0.0021, effect size = 0.12) or between hemispheres (*F*(1, 25) = 1.22, *p* = 0.28, RH mean = 0.0075, LH mean = 0.1224, effect size = 0.06) and no interaction (*F*(1, 25) = 0.87, *p* = 0.36). There were also no effects of the oddball stimulus (Figure 7) between groups (*F*(1, 25) = 0.87, *p* = 0.36, meditator mean = 0.0759, control mean = 0.1409, effect size = 0.34) or between hemispheres (*F*(1, 25) = 0.35, *p* = 0.56, RH mean = 0.1340, LH mean = 0.0757, effect size = 0.31) and no interaction (*F*(1, 25) = 2.13, *p* = 0.16). 

### 3.8. Resting-State Functional Connectivity 

**Figure 8:**
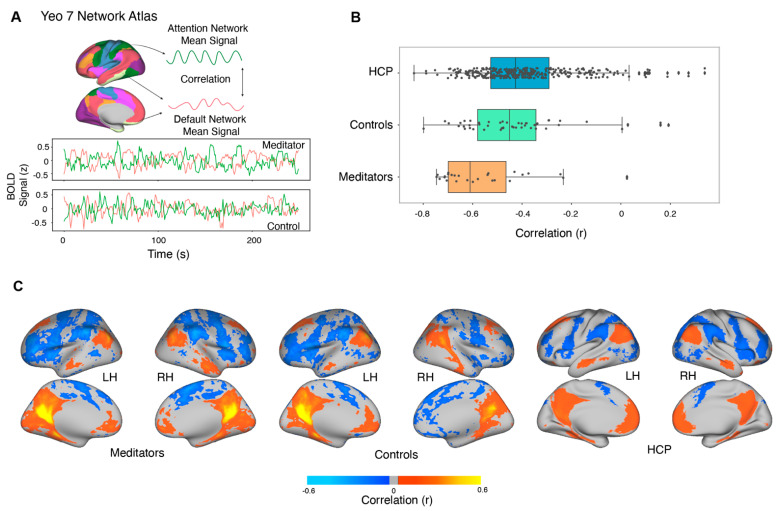
Resting-state functional connectivity (rsFC) of Dorsal Attention Network (DAN) and Default Mode Network (DMN) for the meditator dataset with other non-meditator datasets (**A**) We used the Yeo–Buckner 7 network atlas (Yeo et. al. 2011) to extract the DAN and DMN ROIs; the mean grayordinate signal regressed BOLD signal was averaged across the ROI and correlated across the networks. The DAN and DMN signal is illustrated for a single meditator (VMC) and control subject. (**B**) The meditation group was compared with 168 subjects from the Human Connectome Project and 21 control subjects that included eight sham expertise subjects and thirteen other subjects acquired on the same scanner (Brissenden et. al. 2016). The meditation group had stronger anti correlations as compared to the controls and the HCP dataset subjects, suggesting that the meditation practice brings long-term changes in functional connectivity in the DAN and DMN regions. (**C**) Posterior cingulate cortex (PCC) seed in the left hemisphere was used to create subject averaged connectivity maps in the three datasets.

In order to further examine the group-level difference in DAN–DMN activation differences in the MOT task, we performed resting-state connectivity analysis. In addition to examining the focused attention meditation experts and sham-expertise control subjects, we included additional control subject populations to broaden our analysis and to overcome limitations with attrition in our original subject pool. We acquired two runs (278 timepoints) of resting state data in 13 of our total 16 meditators, and we limited our rsFC analysis to these 13 subjects. We compared the rsFC between DMN and DAN of meditation subjects with the connectivity of eight subjects from the sham-expertise control group and 13 participants acquired on the same scanner with the same parameters analyzed elsewhere [77]. Collectively, we refer to these 21 control subjects as “in-lab” controls. We also compared the connectivity with 168 subjects from the Human Connectome Project (HCP) [51]. Data across multiple resting state runs was normalized and concatenated after minimal preprocessing [78], and mean grayordinate signal was regressed out before the connectivity analysis. We computed the connectivity separately for the left and right hemispheres and collated them in our analysis. There were no differences across the left and right hemispheric connectivity in all the datasets (*F*_(1, 398)_ = 0.032, *p* = 0.85). The meditation group demonstrated greater anticorrelation between DAN and DMN (M = −0.55, SD = 0.18) as compared to the in-lab controls (M = −0.41, SD = 0.21; *t*_(66)_ = −2.72, *p* = 0.008, d = 0.67) and to the HCP dataset (M = −0.39, SD = 0.19; (t_(360)_ = −4.08, *p* < 0.0001, d = 0.83)) (Figure 8). Conversely, there was no statistical difference (t_(376)_ = 0.61, *p* =0.53, d = 0.1) between the HCP dataset and in-lab controls. 

## 4. Discussion

We investigated activation in cortical attention networks while meditators and matched controls conducted two demanding attention tasks. In a sustained attention task, meditators demonstrated a greater separation between activity in the dorsal attention and default mode networks while sustaining attention, relative to controls. Conversely, no differences were observed between meditators and controls during an attention capture task. Resting-state analysis also revealed stronger anticorrelations between Dorsal Attention and Default Mode Networks in experienced meditators than in an in-lab control group and in the larger HCP control group. Although experienced “focused attention” meditators did not exhibit performance benefits on either visual attention task relative to controls, the functional separation between dorsal attention and default mode networks is noteworthy, as prior studies have suggested that strong opposition between the default mode and task-positive networks is associated with greater brain health [79]. 

### 4.1. MOT: Greater DMN Suppression in Meditators While Attending

Our strongest task fMRI finding was the stronger opponency between DAN activation and DMN deactivation in meditators than in sham-expertise controls. Examination of only the DAN revealed a trend-level group effect with a medium effect size (*p* = 0.08, effect size = 0.65). This suggests that our study was somewhat underpowered to observe DAN increases for mediators. However, examination of only the DMN revealed a significant group difference. In the absence of a given task, the mind tends to meandering, self-centered thoughts [41,80,81,82]. Mind wandering and self-related thoughts are correlated with increased activity in the Default Mode Network [83,84,85,86,87,88]. Brief mindfulness training has been shown to reduce mind wandering [89] and alter connectivity between nodes of the DMN both at rest [38] and while in meditation [90,91]. Our current finding that the DMN is more suppressed in meditators while they are actively paying attention builds on these previous findings. Based on prior findings, meditators would be expected to spend less time “in default” then average, certainly at least during the time they spend in active meditation practice (an average of 120 min per day for our participants), and our results suggest that intentional DMN suppression during meditation practice could transfer to other attentionally demanding tasks outside of meditation. 

Behaviorally, we failed to replicate a common finding in meditation studies: improved performance on tasks designed to measure sustained attention (but see [92]), and instead demonstrated an intriguing decoupling of behavior and BOLD activation in meditators, but not controls, while attending. Typically, when performing a multiple object tracking task, as a person’s *k* score (a measure of how many objects they are able to attend to) increases, BOLD activity in the dorsal attention network, particularly the intraparietal sulcus, also increases, both within subjects as attentional load increases [46,53,93,94] and across subjects as *k* score varies [95]. In the current study, we see this expected variation in controls, who demonstrated a strong correlation between *k* score and both DAN activation and DMN suppression. No such relationship existed in the meditators. Although the N is not large in this study, the failure to observe the expected relationship between BOLD signal and number of attentionally tracked objects could reflect diverse effects of training on attention networks across the meditator group. Additionally, since the early stages of meditation training specifically encourage single-pointed attention on a particular object of interest, asking meditators to divide their attention over four objects was perhaps not an optimal way to assess the attentional benefits of meditation training. A parametric variation of the number of tracked items could prove useful in future studies.

### 4.2. ViNO: No Differences in Attentional Capture Associated with Meditation

Our attention capture task combined task-relevant spatial reorienting with infrequent, task-irrelevant oddball stimuli. In order to produce a strong attentional capture effect, we employed highly vivid and novel (shown only once) stimuli. We had hypothesized that experienced focused attention meditators would be more effective in filtering out task-irrelevant distractors in an attentional capture situation, operationalized as less cortical reactivity in the Ventral Attention Network, including the temporoparietal junction, in response to unpredicted stimuli. 

Our findings did not support this hypothesis. Meditators did not display differences in either reaction time when reorienting spatially to an unexpected target, or in cortical activation in the ventral attention network when interrupted by task-irrelevant salient oddball distractors. 

Curiously, experienced meditators exhibited longer RTs overall, relative to the non-meditators. While RTs are longer for meditators overall, this is not indicative of a longer time to reorient. The invalid vs. valid difference is the same across groups, i.e., there is no difference in time to reorient and respond between groups. This reflects a possible difference in strategy between the two groups: while all participants were instructed to “respond as quickly as possible without sacrificing accuracy”, the high accuracy across groups (89% correct in both groups) leaves open the possibility of a speed-accuracy tradeoff that is not detectable in this dataset. 

We hypothesized that the oddball response (i.e., a prediction error signal) would be reduced in the meditation population relative to the controls. It has more recently been suggested that the oddball response may be sufficiently evolutionarily ingrained to remain unmalleable by meditation training [96]. Here, we observe decreased TPJ responsively to oddball stimuli with a small (0.34) effect size. Due to the low temporal resolution of fMRI, an EEG experiment testing the P300 response in meditators (e.g., [97]) using this same task would be an effective way to follow up on this initial trend finding.

### 4.3. Resting-State Functional Connectivity: Experienced Meditators Exhibit Greater DAN–DMN Opponency

Our resting-state functional connectivity analysis of DAN–DMN coupling mirrored our observations during the MOT sustained attention task. Experienced meditators had stronger opponency, in the form of anti-correlated activation, between DAN and DMN than did our in-lab controls. We observed the same result when comparing meditators to subjects from the large, publicly available Human Connectome Project dataset. Our in-lab controls did not differ significantly from the HCP population. Bauer et al. (2019) [98] observed a similar resting-state finding between the DAN and the Central Executive Network, another task-positive network that is distinct from the DAN. Our results expand on this prior finding by demonstrating that the strength of opponent coupling between the DMN and either of the two primary task-positive networks is enhanced in experienced meditators as compared to controls.

## 5. Conclusions

Here, we have investigated trait-level differences between meditators and non-meditators under highly demanding attentional conditions and at rest. We have shown that meditation experience correlates with greater difference between activation in the DAN and the DMN, both when attention must be sustained and during rest. We did not observe any behavioral benefits in either a sustained attention task or in an attention capture paradigm for experienced “focused attention” meditators. Our work adds to a growing literature that long-term meditation practice contributes to brain health and mental wellness by gaining effective suppressive control over the ruminative DMN. While not all mind wandering is ruminative [99], the primary findings are consistent with positive effects of meditation training on suppression of the DMN. Promising topics for future studies include longitudinal, pre- and post-training studies of the effects of meditation on the brain’s attentional networks (e.g., [98]).

## Figures and Tables

**Table 1:** Behavioral data for the Multiple Object Tacking task in meditation and control participants, showing group means ± SEM for hit rate, false alarm rate, and *k* Score.

MOT Behavioral Data			
Group	Hit Rate	False Alarm	*k* Score
Meditation	0.64 ± 0.04	0.33 ± 0.06	1.25 ± 0.32
Control	0.57 ± 0.05	0.39 ± 0.05	0.82 ± 0.22

## Data Availability

The data presented in this study are available on request from the corresponding author. The data are not publicly available due to participant privacy. The Human Connectome Project (HCP) dataset is publicly available on db.humanconnectome.org.

## Appendix A

List of the HCP dataset (db.humanconnectome.org; accessed 26 August 2019) subjects as used in the analysis

100610, 102311, 102816, 104416, 105923, 108323, 109123, 111514, 114823, 115017, 115825, 116726, 118225, 125525, 126426, 128935, 130114, 130518, 131217, 131722, 132118, 134627, 134829, 135124, 137128, 140117, 144226, 145834, 146129, 146432, 146735, 146937, 148133, 150423, 155938, 156334, 157336, 158035, 158136, 159239, 162935, 164131, 164636, 165436, 167036, 167440, 169040, 169343, 169444, 169747, 171633, 172130, 173334, 176542, 177140, 177645, 177746, 178142, 178243, 178647, 180533, 181232, 182436, 182739, 185442, 187345, 191033, 191336, 191841, 192439, 192641, 193845, 195041, 196144, 197348, 198653, 199655, 200311, 200614, 201515, 203418, 204521, 205220, 209228, 212419, 214019, 214524, 221319, 233326, 239136, 246133, 249947, 251833, 257845, 263436, 283543, 318637, 320826, 330324, 346137, 352738, 360030, 365343, 380036, 381038, 385046, 389357, 393247, 395756, 397760, 401422, 406836, 412528, 429040, 436845, 463040, 467351, 525541, 541943, 547046, 562345, 572045, 573249, 581450, 601127, 617748, 627549, 638049, 644246, 654552, 671855, 680957, 690152, 706040, 724446, 725751, 732243, 751550, 757764, 765864, 770352, 771354, 782561, 783462, 789373, 814649, 818859, 825048, 826353, 833249, 859671, 861456, 871762, 872764, 878776, 878877, 898176, 899885, 901139, 901442, 905147, 910241, 926862, 927359, 942658, 943862, 951457, 958976

## References

[1] Kabat-Zinn J. (2003). Mindfulness-Based Interventions in Context: Past, Present, and Future. Clin. Psychol. Sci. Pr..

[2] Tang Y.-Y., Ma Y., Wang J., Fan Y., Feng S., Lu Q., Yu Q., Sui D., Rothbart M.K., Fan M. (2007). Short-term meditation training improves attention and self-regulation. Proc. Natl. Acad. Sci. USA.

[3] Lazar S.W., Bush G., Gollub R.L., Fricchione G.L., Khalsa G., Benson H. (2000). Functional brain mapping of the relaxation response and meditation. NeuroReport.

[4] Manna A., Raffone A., Perrucci M.G., Nardo D., Ferretti A., Tartaro A., Londei A., Del Gratta C., Belardinelli M.O., Romani G.L. (2010). Neural correlates of focused attention and cognitive monitoring in meditation. Brain Res. Bull..

[5] Brefczynski-Lewis J.A., Lutz A., Schaefer H.S., Levinson D.B., Davidson R.J. (2007). Neural correlates of attentional expertise in long-term meditation practitioners. Proc. Natl. Acad. Sci. USA.

[6] Valentine E.R., Sweet P.L.G. (1999). Meditation and attention: A comparison of the effects of concentrative and mindfulness meditation on sustained attention. Ment. Health Relig. Cult..

[7] Lutz A., Slagter H.A., Rawlings N.B., Francis A.D., Greischar L.L., Davidson R.J. (2009). Mental Training Enhances Attentional Stability: Neural and Behavioral Evidence. J. Neurosci..

[8] Moore A., Malinowski P. (2009). Meditation, mindfulness and cognitive flexibility. Conscious. Cogn..

[9] MacLean K.A., Ferrer E., Aichele S.R., Bridwell D.A., Zanesco A.P., Jacobs T.L., King B.G., Rosenberg E.L., Sahdra B.K., Shaver P.R. (2010). Intensive Meditation Training Improves Perceptual Discrimination and Sustained Attention. Psychol. Sci..

[10] Izzetoglu M., Shewokis P.A., Tsai K., Dantoin P., Sparango K., Min K. (2020). Short-Term Effects of Meditation on Sustained Attention as Measured by fNIRS. Brain Sci..

[11] Corbetta M., Shulman G.L. (2002). Control of goal-directed and stimulus-driven attention in the brain. Nat. Rev. Neurosci..

[12] Serences J.T., Shomstein S., Leber A.B., Golay X., Egeth H.E., Yantis S. (2005). Coordination of Voluntary and Stimulus-Driven Attentional Control in Human Cortex. Psychol. Sci..

[13] Corbetta M., Patel G., Shulman G.L. (2008). The Reorienting System of the Human Brain: From Environment to Theory of Mind. Neuron.

[14] Greenberg A.S., Esterman M., Wilson D., Serences J.T., Yantis S. (2010). Control of Spatial and Feature-Based Attention in Frontoparietal Cortex. J. Neurosci..

[15] Somers D.C., Sheremata S.L. (2013). Attention maps in the brain. Wiley Interdiscip. Rev. Cogn. Sci..

[16] Michalka S.W., Kong L., Rosen M.L., Shinn-Cunningham B.G., Somers D.C. (2015). Short-Term Memory for Space and Time Flexibly Recruit Complementary Sensory-Biased Frontal Lobe Attention Networks. Neuron.

[17] Jonides J., Yantis S. (1988). Uniqueness of abrupt visual onset in capturing attention. Percept. Psychophys..

[18] Corbetta M., Kincade J.M., Ollinger J.M., McAvoy M.P., Shulman G.L. (2000). Voluntary orienting is dissociated from target detection in human posterior parietal cortex. Nat. Neurosci..

[19] Fox M.D., Corbetta M., Snyder A.Z., Vincent J.L., Raichle M.E. (2006). Spontaneous neuronal activity distinguishes human dorsal and ventral attention systems. Proc. Natl. Acad. Sci. USA.

[20] Weissman D.H., Prado J. (2012). Heightened activity in a key region of the ventral attention network is linked to reduced activity in a key region of the dorsal attention network during unexpected shifts of covert visual spatial attention. NeuroImage.

[21] Patel G.H., Yang D., Jamerson E.C., Snyder L.H., Corbetta M., Ferrera V.P. (2015). Functional evolution of new and expanded attention networks in humans. Proc. Natl. Acad. Sci. USA.

[22] Theeuwes J. (1994). Endogenous and Exogenous Control of Visual Selection. Perception.

[23] Shulman G.L., Fiez J.A., Corbetta M., Buckner R.L., Miezin F.M., Raichle M.E., Petersen S.E. (1997). Common Blood Flow Changes across Visual Tasks: II. Decreases in Cerebral Cortex. J. Cogn. Neurosci..

[24] Raichle M.E., MacLeod A.M., Snyder A.Z., Powers W.J., Gusnard D.A., Shulman G.L. (2001). A default mode of brain function. Proc. Natl. Acad. Sci. USA.

[25] Greicius M.D., Menon V. (2004). Default-Mode Activity during a Passive Sensory Task: Uncoupled from Deactivation but Impacting Activation. J. Cogn. Neurosci..

[26] Buckner R.L., Andrews-Hanna J.R., Schacter D.L. (2008). The Brain’s Default Network. Ann. N. Y. Acad. Sci..

[27] Power J.D., Schlaggar B.L., Petersen S.E. (2014). Studying Brain Organization via Spontaneous fMRI Signal. Neuron.

[28] Yeo B.T.T., Krienen F.M., Sepulcre J., Sabuncu M.R., Lashkari D., Hollinshead M., Roffman J.L., Smoller J.W., Zöllei L., Polimeni J.R. (2011). The organization of the human cerebral cortex estimated by intrinsic functional connectivity. J. Neurophysiol..

[29] Power J.D., Cohen A.L., Nelson S.M., Wig G.S., Barnes K.A., Church J.A., Vogel A.C., Laumann T.O., Miezin F.M., Schlaggar B.L. (2011). Functional Network Organization of the Human Brain. Neuron.

[30] Cole M.W., Reynolds J.R., Power J.D., Repovs G., Anticevic A., Braver T.S. (2013). Multi-task connectivity reveals flexible hubs for adaptive task control. Nat. Neurosci..

[31] Gonzalez-Castillo J., Bandettini P.A. (2018). Task-based dynamic functional connectivity: Recent findings and open questions. NeuroImage.

[32] Spreng R.N., Stevens W.D., Viviano J.D., Schacter D.L. (2016). Attenuated anticorrelation between the default and dorsal attention networks with aging: Evidence from task and rest. Neurobiol. Aging.

[33] Ramírez-Barrantes R., Arancibia M., Stojanova J., Aspé-Sánchez M., Córdova C., Henríquez-Ch R.A. (2019). Default Mode Network, Meditation, and Age-Associated Brain Changes: What Can We Learn from the Impact of Mental Training on Well-Being as a Psychotherapeutic Approach?. Neural Plast..

[34] Esterman M., Rosenberg M.D., Noonan S.K. (2014). Intrinsic Fluctuations in Sustained Attention and Distractor Processing. J. Neurosci..

[35] Lo O.-Y., Halko M.A., Zhou J., Harrison R., Lipsitz L.A., Manor B. (2017). Gait Speed and Gait Variability Are Associated with Different Functional Brain Networks. Front. Aging Neurosci..

[36] Esposito R., Cieri F., Chiacchiaretta P., Cera N., Lauriola M., Di Giannantonio M., Tartaro A., Ferretti A. (2017). Modifications in resting state functional anticorrelation between default mode network and dorsal attention network: Comparison among young adults, healthy elders and mild cognitive impairment patients. Brain Imaging Behav..

[37] Weathersby F.L., King J.B., Fox J.C., Loret A., Anderson J.S. (2019). Functional connectivity of emotional well-being: Overconnectivity between default and attentional networks is associated with attitudes of anger and aggression. Psychiatry Res. Neuroimaging.

[38] Brewer J.A., Worhunsky P.D., Gray J.R., Tang Y.-Y., Weber J., Kober H. (2011). Meditation experience is associated with differences in default mode network activity and connectivity. Proc. Natl. Acad. Sci. USA.

[39] Lazar S.W., Kerr C.E., Wasserman R.H., Gray J.R., Greve D.N., Treadway M.T., McGarvey M., Quinn B.T., Dusek J.A., Benson H. (2005). Meditation experience is associated with increased cortical thickness. NeuroReport.

[40] Taylor V.A., Daneault V., Grant J., Scavone G., Breton E., Roffe-Vidal S., Courtemanche J., Lavarenne A.S., Marrelec G., Benali H. (2013). Impact of meditation training on the default mode network during a restful state. Soc. Cogn. Affect. Neurosci..

[41] Killingsworth M.A., Gilbert D.T. (2010). A Wandering Mind Is an Unhappy Mind. Science.

[42] Travis F., Shear J. (2010). Focused attention, open monitoring and automatic self-transcending: Categories to organize meditations from Vedic, Buddhist and Chinese traditions. Conscious. Cogn..

[43] Vago D.R., Zeidan F. (2016). The brain on silent: Mind wandering, mindful awareness, and states of mental tranquility. Ann. N. Y. Acad. Sci..

[44] Goenka S.N. (1987). The Discourse Summaries of SN Goenka.

[45] Pylyshyn Z.W., Storm R.W. (1988). Tracking multiple independent targets: Evidence for a parallel tracking mechanism. Spat. Vis..

[46] Bettencourt K.C., Somers D.C. (2009). Effects of target enhancement and distractor suppression on multiple object tracking capacity. J. Vis..

[47] Devaney K.J., Rosen M.L., Levin E.J., Somers D.C. (2019). Identification of Visual Attentional Regions of the Temporoparietal Junction in Individual Subjects using a Vivid, Novel Oddball Paradigm. Front. Hum. Neurosci..

[48] Goodman L.A. (1961). Snowball Sampling. Ann. Math. Stat..

[49] Biernacki P., Waldorf D. (1981). Snowball Sampling: Problems and Techniques of Chain Referral Sampling. Sociol. Methods Res..

[50] Boot W.R., Blakely D.P., Simons D.J. (2011). Do Action Video Games Improve Perception and Cognition?. Front. Psychol..

[51] Van Essen D.C., Smith S.M., Barch D.M., Behrens T.E., Yacoub E., Ugurbil K. (2013). The WU-Minn Human Connectome Project: An overview. NeuroImage.

[52] Scholl B.J., Pylyshyn Z.W., Feldman J. (2001). What is a visual object? Evidence from target merging in multiple object tracking. Cognition.

[53] Culham J.C., Cavanagh P., Kanwisher N.G. (2001). Attention Response Functions. Neuron.

[54] Engell A.D., Haxby J.V. (2007). Facial expression and gaze-direction in human superior temporal sulcus. Neuropsychologia.

[55] Tsao D.Y., Livingstone M.S. (2008). Mechanisms of Face Perception. Annu. Rev. Neurosci..

[56] Jovicich J., Czanner S., Greve D., Haley E., van der Kouwe A., Gollub R., Kennedy D., Schmitt F., Brown G., MacFall J. (2006). Reliability in multi-site structural MRI studies: Effects of gradient non-linearity correction on phantom and human data. NeuroImage.

[57] Ségonne F., Dale A., Busa E., Glessner M., Salat D., Hahn H., Fischl B. (2004). A hybrid approach to the skull stripping problem in MRI. NeuroImage.

[58] Dale A.M., Fischla B., Sereno M.I. (1999). Cortical Surface-Based Analysis: I. Segmentation and Surface Reconstruction. Neuroimage.

[59] Fischl B., Dale A.M. (2000). Measuring the thickness of the human cerebral cortex from magnetic resonance images. Proc. Natl. Acad. Sci. USA.

[60] Fischl B., Liu A., Dale A. (2001). Automated manifold surgery: Constructing geometrically accurate and topologically correct models of the human cerebral cortex. IEEE Trans. Med. Imaging.

[61] Fischl B., Salat D.H., Busa E., Albert M., Dieterich M., Haselgrove C., Van Der Kouwe A., Killiany R., Kennedy D., Klaveness S. (2002). Whole brain segmentation: Automated labeling of neuroanatomical structures in the human brain. Neuron.

[62] Fischl B., Salat D.H., van der Kouwe A.J., Makris N., Ségonne F., Quinn B.T., Dale A.M. (2004). Sequence-independent segmentation of magnetic resonance images. Neuroimage.

[63] Fischl B., Sereno M.I., Tootell R., Dale A.M. (1999). High-resolution intersubject averaging and a coordinate system for the cortical surface. Hum. Brain Mapp..

[64] Fischl B., Sereno M.I., Dale A.M. (1999). Cortical Surface-Based Analysis. NeuroImage.

[65] Fischl B., Van Der Kouwe A., Destrieux C., Halgren E., Ségonne F., Salat D.H., Busa E., Seidman L.J., Goldstein J., Kennedy D. (2004). Automatically Parcellating the Human Cerebral Cortex. Cereb. Cortex.

[66] Greve D.N., Fischl B. (2009). Accurate and robust brain image alignment using boundary-based registration. Neuroimage.

[67] Hagler D.J., Saygin A.P., Sereno M.I. (2006). Smoothing and cluster thresholding for cortical surface-based group analysis of fMRI data. Neuroimage.

[68] Eklund A., Nichols T.E., Knutsson H. (2016). Cluster failure: Why fMRI inferences for spatial extent have inflated false-positive rates. Proc. Natl. Acad. Sci. USA.

[69] MathWorks Inc. (2005). MATLAB: The Language of Technical Computing.

[70] Van Dijk K.R.A., Hedden T., Venkataraman A., Evans K.C., Lazar S.W., Buckner R.L. (2010). Intrinsic Functional Connectivity as a Tool for Human Connectomics: Theory, Properties, and Optimization. J. Neurophysiol..

[71] Power J.D., Barnes K.A., Snyder A.Z., Schlaggar B.L., Petersen S.E. (2012). Spurious but systematic correlations in functional connectivity MRI networks arise from subject motion. Neuroimage.

[72] Carp J. (2012). The secret lives of experiments: Methods reporting in the fMRI literature. Neuroimage.

[73] Schaefer A., Kong R., Gordon E.M., O Laumann T., Zuo X.-N., Holmes A.J., Eickhoff S.B., Yeo B.T.T. (2018). Local-Global Parcellation of the Human Cerebral Cortex from Intrinsic Functional Connectivity MRI. Cereb. Cortex.

[74] Virtanen P., Gommers R., Oliphant T.E., Haberland M., Reddy T., Cournapeau D., Burovski E., Peterson P., Weckesser W., Bright J. (2020). SciPy 1.0: Fundamental algorithms for scientific computing in Python. Nat. Methods.

[75] Seabold S., Perktold J. Statsmodels: Econometric and statistical modeling with python. Proceedings of the 9th Python in Science Conference.

[76] Cowan N. (2001). The magical number 4 in short-term memory: A reconsideration of mental storage capacity. Behav. Brain Sci..

[77] Brissenden J.A., Levin E.J., Osher D.E., Halko M.A., Somers D.C. (2016). Functional Evidence for a Cerebellar Node of the Dorsal Attention Network. J. Neurosci..

[78] Glasser M.F., Sotiropoulos S.N., Wilson J.A., Coalson T.S., Fischl B., Andersson J.L., Xu J., Jbabdi S., Webster M., Polimeni J.R. (2013). The minimal preprocessing pipelines for the Human Connectome Project. Neuroimage.

[79] Whitfield-Gabrieli S., Ford J.M. (2012). Default Mode Network Activity and Connectivity in Psychopathology. Annu. Rev. Clin. Psychol..

[80] James W. (1892). Psychology, Briefer Course.

[81] Smallwood J., Schooler J.W. (2006). The restless mind. Psychol. Bull..

[82] Gruberger M., Ben Simon E., Levkovitz Y., Zangen A., Hendler T. (2011). Towards a Neuroscience of Mind-Wandering. Front. Hum. Neurosci..

[83] Gusnard D.A., Raichle M.E. (2001). Searching for a baseline: Functional imaging and the resting human brain. Nat. Rev. Neurosci..

[84] Mason M.F., Norton M.I., Van Horn J.D., Wegner D.M., Grafton S.T., Macrae C.N. (2007). Wandering Minds: The Default Network and Stimulus-Independent Thought. Science.

[85] Johnson S.C., Baxter L.C., Wilder L.S., Pipe J.G., Heiserman J.E., Prigatano G.P. (2002). Neural correlates of self-reflection. Brain.

[86] Goldberg I.I., Harel M., Malach R. (2006). When the Brain Loses Its Self: Prefrontal Inactivation during Sensorimotor Processing. Neuron.

[87] Schneider F., Bermpohl F., Heinzel A., Rotte M., Walter M., Tempelmann C., Wiebking C., Dobrowolny H., Heinze H., Northoff G. (2008). The resting brain and our self: Self-relatedness modulates resting state neural activity in cortical midline structures. Neuroscience.

[88] Andrews-Hanna J.R., Reidler J.S., Sepulcre J., Poulin R., Buckner R.L. (2010). Functional-Anatomic Fractionation of the Brain’s Default Network. Neuron.

[89] Mrazek M.D., Franklin M.S., Phillips D.T., Baird B., Schooler J.W. (2013). Mindfulness Training Improves Working Memory Capacity and GRE Performance While Reducing Mind Wandering. Psychol. Sci..

[90] Josipovic Z., Dinstein I., Weber J., Heeger D.J. (2012). Influence of meditation on anti-correlated networks in the brain. Front. Hum. Neurosci..

[91] Garrison K.A., Zeffiro T.A., Scheinost D., Constable R.T., Brewer J.A. (2015). Meditation leads to reduced default mode network activity beyond an active task. Cogn. Affect. Behav. Neurosci..

[92] Maccoon N.G., MacLean K.A., Davidson R.J., Saron C.D., Lutz A. (2014). No Sustained Attention Differences in a Longitudinal Randomized Trial Comparing Mindfulness Based Stress Reduction versus Active Control. PLoS ONE.

[93] Jovicich J., Peters R.J., Koch C., Braun J., Chang L., Ernst T. (2001). Brain Areas Specific for Attentional Load in a Motion-Tracking Task. J. Cogn. Neurosci..

[94] Drew T., Vogel E.K. (2008). Neural Measures of Individual Differences in Selecting and Tracking Multiple Moving Objects. J. Neurosci..

[95] Fougnie D., Marois R. (2006). Distinct Capacity Limits for Attention and Working Memory. Psychol. Sci..

[96] Laukkonen R., Slagter H.A. (2020). From many to one: Meditation and the plasticity of the predictive mind. PsyArXiv.

[97] Cahn B.R., Polich J. (2009). Meditation (Vipassana) and the P3a event-related brain potential. Int. J. Psychophysiol..

[98] Bauer C.C.C., Whitfield-Gabrieli S., Díaz J.L., Pasaye E.H., Barrios F.A. (2019). From State-to-Trait Meditation: Reconfiguration of Central Executive and Default Mode Networks. Eneuro.

[99] Christoff K., Irving Z.C., Fox K.C., Spreng R.N., Andrews-Hanna J.R. (2016). Mind-wandering as spontaneous thought: A dynamic framework. Nat. Rev. Neurosci..

